# Biomimetic Directional Liquid Transport on a Planar Surface in a Passive and Energy-Free Way

**DOI:** 10.3390/biomimetics10040223

**Published:** 2025-04-03

**Authors:** Qing’an Meng, Zhangcan Li, Jie Pang, Kaicheng Yang, Junjie Zhou

**Affiliations:** College of Aviation Engineering, Civil Aviation Flight University of China, Chengdu 641419, China; leo00415@163.com (Z.L.); kc1730019975@126.com (K.Y.); 15183240257@163.com (J.Z.)

**Keywords:** directional liquid transport, planar surface, biomimetic, passive and energy-free

## Abstract

The development of efficient directional liquid transport systems has become a central focus in numerous research and engineering fields. Natural organisms have evolved intricate structures that facilitate the controlled movement of liquids on planar surfaces. These natural mechanisms offer insights into creating sustainable, energy-efficient technologies that mimic these natural adaptations. The purpose of biomimetic directional liquid transport is to harness the principles found in nature to design systems that can autonomously manage the flow of liquids. One of the core objectives is to achieve efficient liquid directional movement without the need for external energy sources or mechanical pumps. In this article, we review the typical models of natural systems with directional liquid transport on planar surfaces. Next, we reveal the physical mechanism by which surface chemical gradients, wettability gradients, and geometric gradients synergically drive liquid directional motion. Then, we introduce the breakthroughs of bionic surface engineering strategies in water harvesting, directional liquid transport and recent advancements in engineering applications. Finally, we give a conclusion and future perspectives on the development of directional liquid transport.

## 1. Introduction

Over billions of years of evolution, natural systems have developed sophisticated liquid manipulation strategies [1,2,3,4,5,6,7,8,9,10]. Natural surfaces exhibit spontaneous directional liquid transport as an essential survival mechanism, enabling organisms to maintain hydration in extreme environments, as exemplified by the fog-harvesting adaptations of Namib desert beetles [1] and fluid regulation systems in plant leaves [9]. These phenomena emerge from the cooperative interplay between multiscale morphological features, chemical patterning, and surface energy gradients, achieving optimized liquid control through minimized energy expenditure. Current classification systems differentiate directional transport mechanisms based on three fundamental parameters: contact line morphology, advancing–receding contact angle differentials, and substrate curvature characteristics [11,12]. This taxonomy distinguishes between planar and curved surface dynamics while considering macrostructural organization and microscale surface modifications. For instance, the Namib Desert beetle captures fog droplets via alternating hydrophilic–hydrophobic regions on its elytra and guides their directional flow [1]. The Texas horned lizard utilizes geometrically asymmetric microchannels in its skin to generate unidirectional Laplace pressure differences [2]. The pitcher plant achieves rapid liquid film transport through hierarchical wedge-shaped microcavities on its peristome [9]. The spider silk features periodic spindle-knots and joints, where roughness heterogeneity and geometric curvature gradients synergistically overcome hysteresis to propel micrometer-scale droplets toward spindle-knots [6]. The cactus integrates gradient microgrooves, oriented barbs, and trichomes at the spine base, utilizing roughness-induced surface energy gradients and anisotropic contact angle hysteresis to drive rapid droplet transport from the tip to the base [7]. These natural solutions fundamentally rely on optimized balance between fluid adhesion control and flow resistance minimization through synergistic surface energy manipulation, structural anisotropy, and multiscale morphological integration.

In recent years, based on bionic concepts, researchers have developed a variety of advanced planar and curved materials and devices with rich and novel functions to address global challenges such as water scarcity and advancing microfluidic technologies [13,14,15]. Liquids present different wetting behaviors on the surface of the artificial planar and curved advanced materials, enabling various functions, such as water harvesting [16,17,18], self-cleaning [19,20,21], oil/water separation [22,23], heat transfer [24,25], microfluidics [26,27,28], and medical analysis [29].

Although a number of researchers have carried out detailed reviews on bionic liquid directional transport, the available reviews are mostly based on a generalization of all biomimetics of living organisms without a detailed differentiation. Actually, the surface morphologies (planar and curved) presented by the organisms as well as the biomimetic materials determine the mechanism of liquid wetting on the surface, the liquid transport behaviors, and the corresponding applications. However, the existing reports have not yet provided a detailed overview of the directional transport of fluids on organisms and on biomimetic materials based on surface morphologies.

In this paper, we provide a systematic review of bio-inspired directional transport of liquids mainly on planar surfaces in a passive, energy-free manner and their progress in engineering applications. First, the phenomenon of directional liquid transport on the typical natural models (beetles, horned lizards, pitcher plants) is analyzed. Second, we analyze the quantitative model of the synergistic effect of chemical energy gradients, wettability gradients and geometric gradients, and the regulation rules of Laplace pressure, capillary force, and surface tension are revealed. Then, we summarize the latest research achievements of bionic surface engineering strategies in water harvesting and liquid directional transport and their applications in engineering. Finally, current technological challenges and future directions are reviewed.

## 2. Directional Liquid Transport on Natural Planar Systems

**Figure 1:**
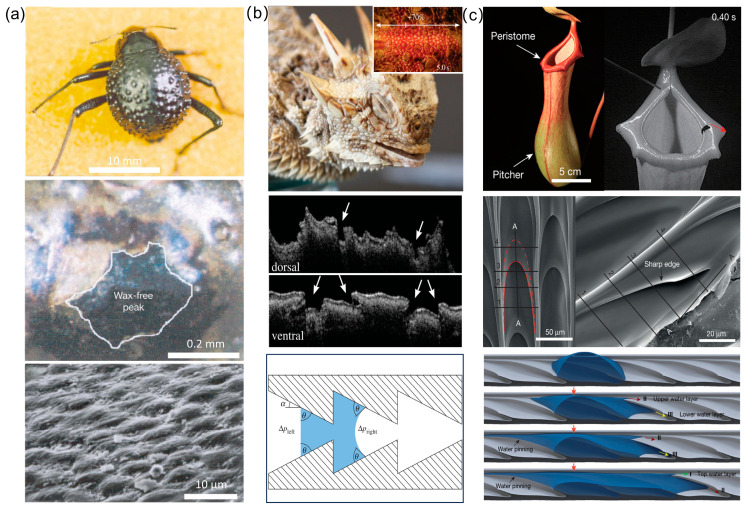
Directional liquid transport on natural planar systems: (**a**) The Namib Desert beetle utilizes hydrophilic and hydrophobic regions on its elytra to collect fog droplets toward the beetle’s mouthparts; (**b**) The Texas horned lizard employs a passive capillary network within its integument to transport water directionally toward its snout. The white arrows indicate the capillaries; (**c**) The carnivorous pitcher plant Nepenthes alata utilizes its peristome—a rim-like structure—to achieve rapid, continuous water transport from the inner to the outer margin. Images reproduced with permission from Refs. [1,2,9].

**Desert Beetles: Hydrophilic–Hydrophobic Patterning**—The Namib Desert beetle survives in hyperarid conditions by harvesting water from fog-laden winds. Its elytra (wing covers) exhibit a unique microstructure comprising alternating hydrophilic and hydrophobic regions [1]. The hydrophilic “bumps” act as nucleation sites for fog droplets, while the surrounding hydrophobic, wax-coated valleys facilitate droplet coalescence and directional rolling toward the beetle’s mouthparts (Figure 1a). This process relies on a critical balance between droplet adhesion and detachment forces. When droplets reach a size where gravitational force overcomes capillary adhesion, they roll downward along the beetle’s tilted body, guided by surface topography. Experimental biomimetic models using hydrophilic–hydrophobic patterned surfaces, such as glass spheres embedded in wax, have replicated this mechanism, achieving efficient fog collection. The beetle’s design demonstrates how microscale wettability gradients and macro-scale tilt can synergize to enable passive, energy-free water harvesting.

**Texas Horned Lizards: Capillary “Liquid Diodes”**—The Texas horned lizard employs a passive capillary network within its integument to transport water directionally toward its snout [2,30,31]. The skin features interconnected microchannels with asymmetric geometries: channels narrow gradually toward the snout but widen abruptly in the reverse direction (Figure 1b). This structural asymmetry generates differential Laplace pressures at liquid–air interfaces, promoting unidirectional flow. Specifically, narrowing channels create concave menisci that drive capillary flow forward, while abrupt widening in the backward direction produces convex menisci that pin the liquid front. Additionally, lateral interconnections between longitudinal channels enable continuous transport by “recycling” pinned liquid through adjacent pathways. Theoretical models based on the Young–Laplace equation and experimental prototypes (e.g., laser-engraved PMMA surfaces) confirm that such geometries act as “liquid diodes”, achieving flow directionality ratios of up to 2:1. This mechanism highlights the role of geometric gradients and network connectivity in overcoming hydraulic resistance and enabling sustained transport without external energy input.

**Pitcher Plants: Multiscale Capillary Architectures**—The carnivorous pitcher plant Nepenthes alata utilizes its peristome—a rim-like structure—to achieve rapid, continuous water transport from the inner to the outer margin [9]. The peristome’s surface comprises hierarchical microgrooves with two-tiered architecture: primary grooves contain secondary grooves lined with overlapping, duck-billed microcavities (Figure 1c). These microcavities feature gradient wedge angles and sharp edges, which enhance capillary rise. Water ascends along the narrowing wedges, while reverse flow is inhibited by pinning at the sharp edges. Crucially, the closed tops of the microcavities allow liquid to overflow into adjacent cavities, creating a multistage pumping effect. Superhydrophilicity further accelerates transport, achieving velocities of ~78 mm/s. Artificial replicas using polydimethylsiloxane (PDMS) confirmed that directional flow depends on both surface chemistry (hydrophilicity) and structural asymmetry, rather than chemical gradients. This system exemplifies how combining geometric gradients with wettability optimization can achieve high-speed, unidirectional transport.

The natural systems reviewed here provide valuable insights into the mechanisms of directional water transport on planar surfaces, all of which employ unique surface structures to optimize water harvesting and transport. These natural systems demonstrate the importance of surface chemistry, microstructure, and geometry in enhancing fluid transport efficiency without any energy input. By decoding these natural blueprints, scientists have developed sustainable technologies for developing innovative materials and devices for water harvesting, directional liquid transport, and other applications.

## 3. Physical Principles and Mechanisms

**Figure 2:**
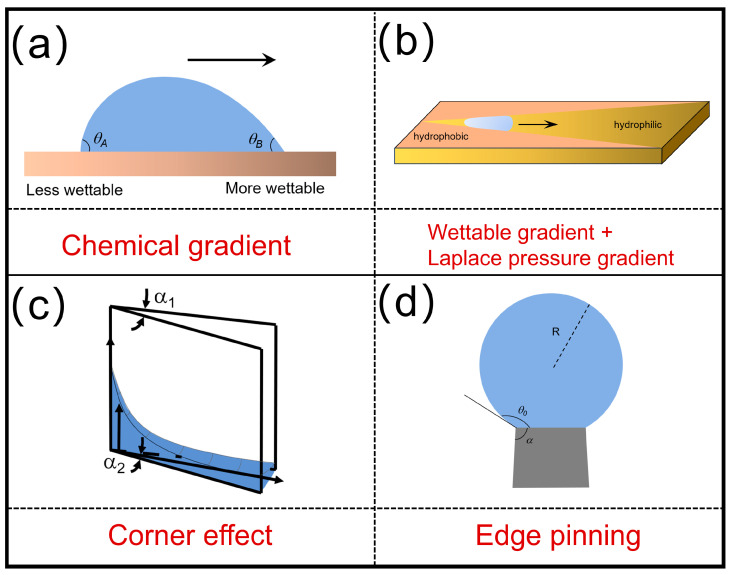
Schematic representation of driving forces exerted on the liquid to transport. (**a**) Anisotropic wetting of droplets on chemical gradient surface; (**b**) Wettable gradient and Laplace pressure gradient on a planar surface combined wedge-shaped pattern; (**c**) Capillary rise under the corner effect; (**d**) Edge pinning effect of a droplet on a sharp edge.

On a planar system, the dynamic wetting behavior of liquid is mainly embodied in water harvesting and directional transport. For water harvesting, the directional motion of liquids on plane solid surfaces is fundamentally driven by the synergistic effects of chemical energy gradients, wettability gradients, and Laplace pressure gradients, rooted in the interplay of interfacial energies at the solid–liquid–gas triple phase boundary. Chemical energy gradients arise from spatial variations in surface chemistry, creating differences in surface free energy that modulate the contact angle (*θ*) via the Young equation:

(1) $$\gamma_{sg}=\gamma_{sl}+\gamma_{lg}\cos\theta \;$$

where *γ_sg_*, *γ_sl_*, and *γ_lg_* represent solid–gas, solid–liquid, and liquid–gas interfacial tensions, respectively. This gradient induces a directional driving force, as liquids migrate toward regions of higher surface energy (e.g., hydrophilic zones) to minimize the system’s free energy. For instance, Chaudhury and Whitesides [32] demonstrated that a surface free-energy gradient constructed by decyltrichlorosilane vapor could propel water droplets (1–2 μL) uphill at speeds of 1–2 mm s^−1^, with the driving force quantified as follows:

(2) $$F_{Chemical}=\pi R_{0}\gamma \left(\cos\theta_{B}-\cos\theta_{A}\right)$$

where *R*_0_ is the droplet radius, *γ* is surface tension, and *θ_B_* (advancing) and *θ_A_* (receding) are contact angles at hydrophilic and hydrophobic regions [33,34], as shown in Figure 2a.

Complementing this mechanism, wettability gradients leverage microstructural patterning or chemical modifications to generate asymmetric contact angle distributions. For a plane surface combined with wettability gradients and Laplace pressure difference generated by a wedge pattern (Figure 2b), the driving force *F_wg_* arising from the wettable gradient can be described as follows:

(3) $$F_{wg}=\alpha L^{2}\gamma k\sin\theta_{M}$$

where α is the half wedge angle, *γ* is the surface tension of water, *L* is the length of the droplet along the wettable gradient direction, *k* is the wettable gradient, and *θ_M_* is the contact angle at the more wettable side (*M*) of the droplet [35,36]. The Laplace pressure force (*F_L_*) in the lengthwise direction generated by the wedge pattern on the wettable gradient surface can be evaluated as follows:

(4) $$F_{L}=A\alpha +B$$

in which *A*, *B* is the constant coefficient for a given volume of a droplet [37,38]. At the same time, the difference of wettability between the inside and the outside of the wedge pattern also provides another driving force (*F_wd_*), which can be described as follows:

(5) $$F_{wd}=2L\gamma \left(\cos\theta_{avg}-\cos\theta_{0}\right)\sin\alpha$$

in which *θ_avg_* is the average contact angle of the droplet over the length of the drop and *θ*_0_ is the contact angle of the hydrophobic area outside the wedge pattern [36,39].

The peristome surface of Nepenthes alata exhibits another directional liquid transport mechanism driven by its unique structural features, particularly the gradient wedge angles and asymmetric confinement effects (Figure 2c). The arch-shaped microchannels enable unidirectional water transport against gravity, attributed to the interplay between capillary forces and gravitational equilibrium. The equilibrium height He(x) of water in a gradient wedge is derived from energy minimization, balancing interfacial and gravitational potentials as follows:

(6) $$H\left(x\right)=\frac{2\gamma \cos\theta }{\rho gx\alpha_{1}}+\frac{\alpha_{1}-\alpha_{2}}{\alpha_{1}h}H^{2}\left(x\right)\;\rho$$

where *γ* is the liquid surface tension, *ρ* is the liquid density, *θ* indicates the contact angle, *α* is the opening angle, *g* is the gravitational acceleration, *h* is the height of the intersecting plates, and *H*(*x*) is the height of the liquid surface at position *x* [9].

At the sharp solid edges, a liquid exhibits special wetting behaviors (Figure 2d). At the solid−liquid−gas three-phase contact line, a liquid droplet is pinned by the solid sharp edge and prevented from further moving [40,41]. The relationship between the apparent contact angle (*θ*_0_) of a liquid at the edge of a solid and its inherent contact angle (*θ*) can be described as follows:

(7) $$\theta_{0}\leq \pi -\varphi +\theta$$

where φ is the angle between solid edges [40,42].

Water harvesting and directional liquid transport exhibit both connections and distinctions in their physical mechanisms and surface structures. In terms of physical mechanisms, both leverage gradient effects to drive directional liquid motion, yet their implementations differ: water harvesting relies on surface energy gradients (from superhydrophilic/superhydrophobic regions) and Laplace pressure gradients to promote droplet aggregation, whereas directional liquid transport utilizes synergistic capillary effects (via microgrooves) and pinning effects (from arc-shaped concave pits) to achieve rapid unidirectional flow while suppressing backflow. For surface structures, both systems adopt multiscale designs, but water harvesting focuses on macroscopic patterned surfaces (e.g., hydrophilic zones on hydrophobic substrates), while directional transport emphasizes microscale continuous architectures (e.g., tilted grooves with arc-shaped pits).

## 4. Directional Water Harvesting on Biomimetic Systems

The global freshwater scarcity has motivated scientific communities to develop advanced water harvesting systems through biomimetic approaches. This research direction derives inspiration from multi-organism hydraulic regulation mechanisms: the wettability heterogeneity observed in Namib Desert beetle dorsa [43,44,45,46], the morphological gradient inherent in spider silk spindle-knots [36,47], the asymmetric microchannel architecture of Texas horned lizard skin [2], and the multiscale structures of Nepenthes peristomes [48,49] have collectively informed the creation of high-performance atmospheric water harvesters. These bioinspired systems attain superior water capture efficiency through synergistic coordination of surface wettability modulation and geometric parameter optimization. While conventional homogeneous surfaces exhibit limited performance due to droplet adhesion phenomena [36,50,51], nature-derived designs demonstrate enhanced hydraulic management via microscale chemical and topological differentiation.

**Figure 3:**
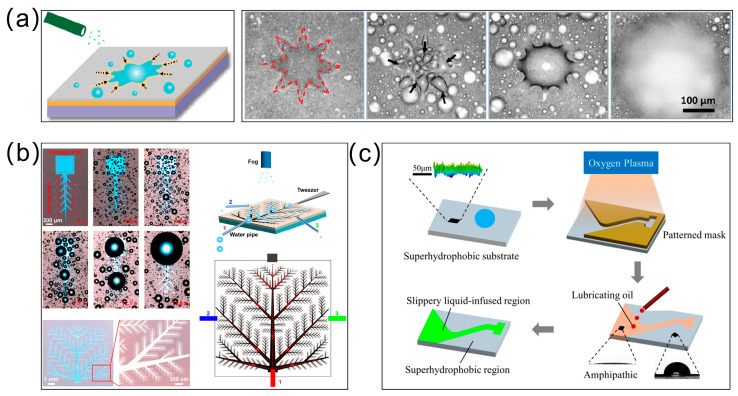
Water harvesting on biomimetic planar surfaces. (**a**) Water harvesting on bioinspired wettability gradient pattern surface with a star-shaped hydrophilic architecture on superhydrophobic TiO_2_ substrates; (**b**) Tiny water droplet collection on a branch of tree-shaped hierarchical cones fabricated on a superhydrophobic surface; (**c**) A superhydrophobic region and a hydrophobic region with infused lubricating oil. Reproduced with permission from Refs. [36,50,58].

The field has witnessed substantial progress in surface engineering methodologies for moisture harvesting applications. Three principal strategies have emerged as particularly effective: Primarily, patterned wettability surfaces demonstrate remarkable potential. Water harvesting saw breakthroughs with Xu’s beetle-inspired membrane [52], collecting 1043 mg·cm^−2^·h^−1^ fog via wettability gradients (CA = 12°–158°). Bai et al. [36] implemented stellate hydrophilic architectures on superhydrophobic TiO_2_ substrates through UV lithography, capitalizing on contact angle asymmetry and Laplace pressure gradients induced by wedge structures. This configuration realized a moisture collection capacity of 2.78 g/cm^2^·h using octagram patterns, representing a 68% enhancement compared to conventional circular designs (Figure 3a). In parallel, Zhu et al. [43,45] developed a complementary inverse configuration where hydrophobic domains accelerated droplet coalescence and detachment, while hydrophilic pathways enabled directional liquid film transport, attaining an exceptional efficiency of 1316.9 mg·h^−1^·cm^−2^ (Table 1).

Alongside chemical patterning approaches, physical modification techniques present alternative solutions. Non-chemical methods, including femtosecond [44,53] and picosecond [46] laser direct writing have been successfully applied for precision wettability engineering. A representative study by Zhang et al. [53] demonstrated titanium surfaces with periodic square arrays achieving 1400 mg·h^−1^·cm^−2^ fog collection efficiency, doubling the performance of untreated substrates. While laser processing offers distinct advantages in resolution and scalability [44,46], persisting challenges in cost-effective mass production through process optimization require further investigation [54]. This technological gap highlights the necessity of continued research into scalable manufacturing protocols that maintain precision while reducing unit costs.

Geometric configuration optimization serves as a fundamental determinant in water harvesting performance enhancement. This principle of structural biomimicry has been systematically extended through hierarchical architecture design. Wang et al. [50] fabricated dendritic conical arrays on superhydrophobic TiO_2_ substrates via laser direct writing, exploiting coordinated surface energy modulation and Laplace pressure amplification to elevate collection efficacy by 36-fold relative to planar surfaces. The multiscale branching configuration not only expanded effective droplet nucleation areas but also promoted rapid coalescence and detachment through apex curvature gradients (Figure 3b). Complementing these vertical hierarchies, horizontal transport optimization has yielded comparable breakthroughs. Lin et al. [47] implemented a quadri-level wedge-channel system with Cu(OH)_2_ nanowire decoration, synergizing capillary forces and curvature-driven propulsion mechanisms to realize a collection rate of 467.30 mg·cm^−2^·h^−1^—an 11.5-fold improvement over untreated surfaces. The frontier of fluid dynamics manipulation has further propelled performance limits. Yan et al. [52,55] pioneered serial cycloid (SSCP) and accelerated brachistochrone (ASBP) patterns, refining contact line geometry and junction transitions to achieve droplet velocities of 289 mm/s and 158 mm/s, respectively. Notably, SSCP minimizes flow resistance through periodic curvature adaptation, while ASBP enhances gravitational energy conversion efficiency [55].

The technological evolution has catalyzed cross-disciplinary implementation. Zhu et al. [56] incorporated TiO_2_ photocatalytic functionality into wedge-patterned interfaces, attaining concurrent water production (14.9 mg·min^−1^·cm^−2^) and pesticide degradation capabilities for enhanced water safety. In thermodynamic system optimization, Hou et al. [57] engineered superhydrophilic triangular microarrays that achieved 110.7 mg·cm^−2^·h^−1^ dew collection under minimal subcooling conditions (ΔT = 2 °C). Device flexibility has emerged as a critical expansion vector: Liu et al. [58] validated 2.51% water harvesting efficacy on lubricant-infused PDMS substrates at 25° inclination, demonstrating compatibility with complex 3D curvatures (Figure 3c). Emerging hybrid systems reveal multifaceted potential: Microfluidic integration [59,60,61] enables precise flow control, while agricultural prototypes like Hu et al.’s PVDF-HFP/Cu(OH)_2_ membrane [62] successfully sustained mint cultivation for 5 days without external irrigation. Zhang et al. [61] further innovated by coupling photovoltaic elements with water harvesters, achieving dual-output generation (1.2 L/m^2^·h water and 15.6 W/m^2^ electricity). These diverse implementations underscore the technology’s versatility across environmental, agricultural, and energy sectors.

Despite transformative progress, critical challenges demand resolution. Scalable manufacturing of intricate architectures necessitates cost-effective techniques such as roll-to-roll nanoimprinting [54] or template-assisted replication [53]. Material durability under extreme operational conditions (e.g., cryogenic temperatures, hypersaline environments) mandates advanced modifications including fluoropolymer coatings [44] or dynamic covalent network polymers [57]. Looking forward, intelligent system development represents the next evolutionary phase: Implementation of stimuli-responsive wettability switching mechanisms (light/thermal/magnetic actuation [55,59]) could enable autonomous environmental adaptation. Contemporary bioinspired water harvesting systems have demonstrated unprecedented capabilities through multiscale structural innovation, dynamic interfacial control, and multifunctional integration. Future research priorities should emphasize scalable fabrication protocols [53,54], extreme-condition durability enhancement [44,57], and smart responsiveness implementation [55,59] to deliver sustainable hydrologic solutions for arid ecosystems.

**Table 1:** Water harvesting efficiency on different planar structures.

Preparation Method	Structure	Water Harvesting Efficiency	Ref.
Photolithography	star-shaped	2.11–2.78 g cm^−2^ h^−1^	[36]
Inkjet Printing	superhydrophilic-superhydrophobic surfaces	1316.9 mg cm^−2^ h^−1^	[43]
Femtosecond Laser	micro/nanopatterns	200 mg cm^−2^ h^−1^	[44]
Chemical Deposition	wedge-shaped tracks	467.30 mg cm^−2^ h^−1^	[47]
Laser Ablation	superhydrophilic-superhydrophobic surfaces	97 mg cm^−2^ h^−1^	[54]
Laser Ablation	superhydrophilic patterns	14.9 ± 0.2 mg min^−1^ cm^−2^	[56]
Laser Ablation	Superhydrophilic-superhydrophobic surfaces	110.7 ± 5.7 mg cm^−2^ h^−1^	[57]

## 5. Directional Liquid Transport on Biomimetic Systems

Natural systems have inspired artificial surfaces capable of spontaneous, rapid, and long-distance liquid transport without external energy input [63,64,65,66,67,68,69,70,71,72]. This evolutionary design paradigm has been concretized through multiple architectural implementations: Capillary networks inspired by reptilian integumentary systems [68,70,73] incorporating periodic hydrophilic/hydrophobic striations demonstrated directional aqueous transport in arid environments with a propulsion velocity of 3.2 mm/s—nearly double conventional benchmarks [68] (Table 2). Through synergistic integration of 3D-printed microconduits with optimized curvature radii, these biomimetic architectures markedly enhanced droplet adaptability across topographically complex substrates [73].

**Figure 4:**
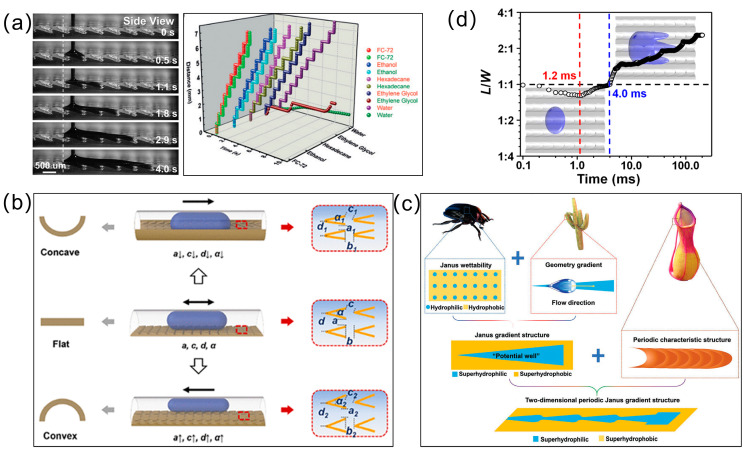
Directional liquid transport on biomimetic planar surfaces. (**a**) Liquid unidirectional transportation on peristome-mimic surface with surface tensions ranging from 9.5 to 72.0 mN m^−1^; (**b**) Transformation of liquid transport direction on the V-shaped prisms. The moving directions of liquid reverse with varying arrangements of the V-shaped prisms on the elastic film; (**c**) Directional water transport on planar surface with a periodic Janus gradient structure inspired from the Janus wettability surface of desert beetle, the geometry gradient of cactus spines, and the periodic microcavities of Nepenthes alata; (**d**) The variation of the ratio between the spreading length and the spreading width as a function of spreading time. Reproduced with permission from Refs. [59,65,69,80].

Contemporary research focuses on four principal operational paradigms: capillary gradient propulsion, wettability modulation, dynamic topological reconfiguration, and multiphysics coupling mechanisms. Among these, capillary differential systems dominate current innovation trajectories: asymmetric geometric constructs (e.g., wedge grooves, arc cavities) generate Laplace pressure differentials through interfacial curvature manipulation [74,75,76,77,78]. Li et al. [65] engineered a unidirectional fluid transport system mimicking Nepenthes alata peristomes, resolving intrinsic limitations of passive fluid control in transport range, energy autonomy, and structural symmetry constraints. Utilizing micro-stereolithography, they fabricated arch-shaped microcavities with acute rear overhangs, achieving spontaneous liquid propulsion across surface tensions (γ = 9.5–72.0 mN/m) and viscosities (η = 0.3–96.0 mPa·s) at velocities up to 50 mm/s (Figure 4a). Complementing botanical models, zoological inspirations yield comparable breakthroughs: Wang et al. [66] designed duck-billed microcavities replicating carnivorous plant morphology, where frontal curvature radii and submicron rear overhangs enabled ethanol transport at 50 mm/s.

Wettability gradient engineering represents a parallel innovation axis: Wu’s team [79] implemented UV-degradable TMOS coatings to create dynamic wetting patterns, achieving precision droplet displacement through photolithographic stripe modulation. Emerging responsive systems bridge static and dynamic control: Li et al. [59] developed curvature-actuated V-shaped prism microarrays (VPMs) that reversibly adjusted transport direction via mechanical deformation, accelerating acid–base neutralization kinetics by 40%. The PDMS-based VPMs modulated contact angle hysteresis through programmable curvature variations as shown in Figure 4b. Chemical homogeneity challenges have been creatively addressed: Xu et al. [60] engineered adhesion-contrast surfaces using honeycomb (HC) and pin-cushion (PC) microstructures, where droplets preferentially migrated toward PC regions due to reduced contact line pinning.

**Table 2:** The speed of directional liquid transport on different planar structures.

Preparation Method	Structure	Directional Liquid Transport Speed	Ref.
Stereolithography	microcavity-Arrayed Surface	50.0 mm/s	[65]
UV Lithography	microgrooves	50.0 mm/s	[66]
Lithography	microstructure	1 mm/s (uphill)	[67]
Laser Engraving	capillary channel network	1 mm/s (average)	[68]
Vapor Deposition and Chemical Etching	janus gradient structure	over 400 mm/s	[69]
3D Printing	microgrooves	50.0 mm/s (average)	[71]
Photolithography and UV Exposure	wettability-patterned surfaces	1 mm/s (uphill)	[72]
3D Printing	topological surface	50.0 mm/s (average)	[80]
3D Printing	microstructure array	23 mm/s	[81]
Thermal Deposition	wedge-shaped	47.6 mm/s (average)	[82]

Optimizing transport efficiency necessitates balanced interfacial energetics: Structural refinement via DLP 3D-printed A-shaped islands suppressed secondary flow vortices, elevating hexadecane velocity to 6 mm/s [83]. Wettability matching theory postulates that maximum momentum transfer occurs when γ_S_/γ_L_ ≈ 1 [84], a principle exemplified by Xie’s periodic Janus gradient structure (PJGS). Through synergistic wedge-arc units, PJGS attained droplet velocities of 400 mm/s (Figure 4c), attributed to aligned surface energy gradients and curvature coordination [69]. Dynamic wetting analysis further elucidates transport mechanisms: Yu et al. [80] investigated the dynamic wetting behavior of liquid droplets on solid surfaces, focusing on the regulatory mechanism of multiscale curvature on superhydrophilic surfaces during droplet impact. Inspired by the peristome surface of Nepenthes alata, the authors fabricated a biomimetic polyvinyl alcohol (PVA) surface with hierarchical curvature structures through 3D-printed mold replication and ethanol-induced shrinkage. Experimental results revealed time-dependent spreading dynamics: bidirectional axial spreading dominated by inertia transitioned to unidirectional circumferential spreading driven by capillary effects (Figure 4d). Theoretical analysis demonstrated that the synergy between air-cushion dynamic pressure balance and capillary forces combined with multiscale curvature modulation of inertia-capillary timescales governed this behavior.

Technological applications span interdisciplinary domains: in microfluidic systems, Liang’s arrowhead microstructures [81] enhanced Y-mixer efficiency fourfold while reducing pressure drop by 35%, enabling high-throughput protein crystallization screening. Biomedical integration demonstrates clinical potential: Gao’s lizard-inspired microfluidic chip integrated electrochemical sensors for chronic wound monitoring, achieving 6% pH sensitivity and TNF-α detection accuracy (R^2^ = 0.9958) [85]. Multiphysics hybridization expands design frontiers: Zhang’s team [86] integrated superhydrophobic copper mesh (CA = 156°) with quartz tubes (CA = 32°), exploiting curvature-induced Laplace pressure to drive underwater oil ascent against gravity over 46 mm.

Despite progress, challenges persist in dynamic response speed [33,87], multiphase flow control [88,89], and long-term durability [73,90]. Current shape-memory materials’ response times hinder real-time microfluidic applications [33], while complex fluids (e.g., blood, non-Newtonian liquids) demand further mechanistic exploration [88]. Future directions include stimuli-responsive materials (photo/thermal/magnetic) [84,91], hybrid manufacturing (ALD-3D printing) [83,92], and machine learning-driven optimization [82,93]. Notably, Bai’s origami structure [82] inspired by scallop locomotion achieved 450 mL·h^−1^ flux via periodic bending, offering novel solutions for wearable fluidics [94]. As biomimetic design converges with nanotechnology, directional liquid transport systems will unlock transformative applications in precision medicine and sustainable energy.

## 6. Engineering Applications and Innovations

**Figure 5:**
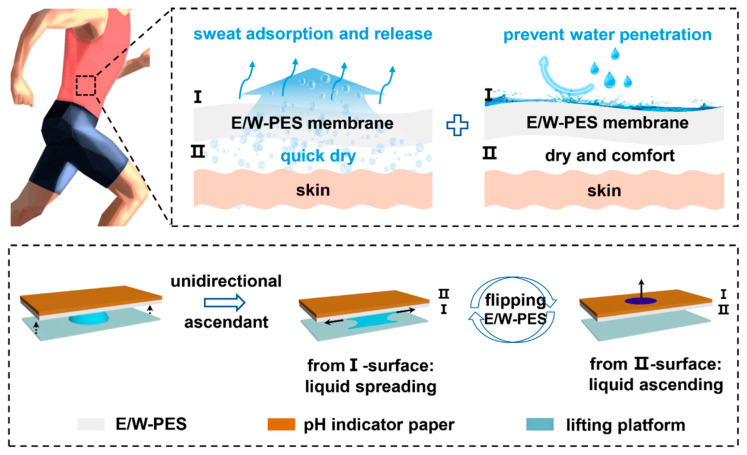
The antigravity unidirectional liquid transport in the porous PES membrane aimed at achieving optimal comfort through the efficient drainage of excess sweat, demonstrating enhanced moisture permeability and superior moisture-wicking capabilities. Reproduced with permission from Ref. [96].

***Smart fabrics*:** Biomimetic textile engineering has emerged as a transformative paradigm for developing functional fabrics with programmable fluidic behavior. Recent advances demonstrate remarkable progress in fabricating intelligent textiles through nature-inspired wettability modulation. As a representative example, Zhou et al. engineered Janus fabrics by implementing unilateral 254 nm UV irradiation on fluoro-polymer/silica-coated polyester substrates, establishing precisely controlled wettability gradients. This architecture attained surface-tension-selective transport capabilities (breakthrough pressure: 1.27 kPa) for diverse liquids (water/soybean oil/hexadecane), with thermal healing at 130 °C enabling full functionality restoration [95]. Building upon vascular-inspired designs, hierarchical pore architectures have expanded directional fluid control. Zhang’s team developed monolithic polyethersulfone (PES) membranes featuring pore size gradients (50–200 nm), which facilitated pH-independent (1–14) unidirectional penetration through Laplace pressure-hydrostatic pressure equilibrium manipulation. Achieving a 16 cm water column breakthrough pressure, this innovation shows particular promise for advanced wound dressing applications [96], as shown in Figure 5.

Converging multiple natural principles enhances textile performance synergistically. Liu et al. integrated desert beetle wettability contrast with cactus spine morphology to create directional sweat-transporting knits. The tree-shaped fluidic pathways, formed through enclosed hydrophobic/hydrophilic gradient patterning, demonstrated exceptional moisture permeability, making them ideal for high-performance sportswear [97]. Extending this concept, Kumar optimized anti-gravity liquid transport through 3D chemical wettability gradients in fluorine-free nanocomposite coatings. By leveraging Laplace pressure-driven pinning–depinning dynamics, the system achieved a 6.7 cm breakthrough pressure, significantly outperforming conventional homogeneous materials [98].

Innovative multifunctional integration represents the frontier of smart textile development. Liu et al. [99] pioneered electrosprayed PDMS/PMMA patterned surfaces enabling dual functionality: directional droplet manipulation along programmable C/S-shaped trajectories and binary signal encoding through droplet positioning. Remarkably, these textiles maintained >99% oil–water separation efficiency across 10 operational cycles while executing complex fluidic logic operations. Collectively, these biomimetic strategies underscore the transformative potential of intelligent textiles in addressing critical challenges in personal moisture management, medical protective equipment, and environmental remediation technologies. The field continues to evolve through cross-disciplinary integration of microfluidics, responsive materials, and sustainable manufacturing principles.

**Figure 6:**
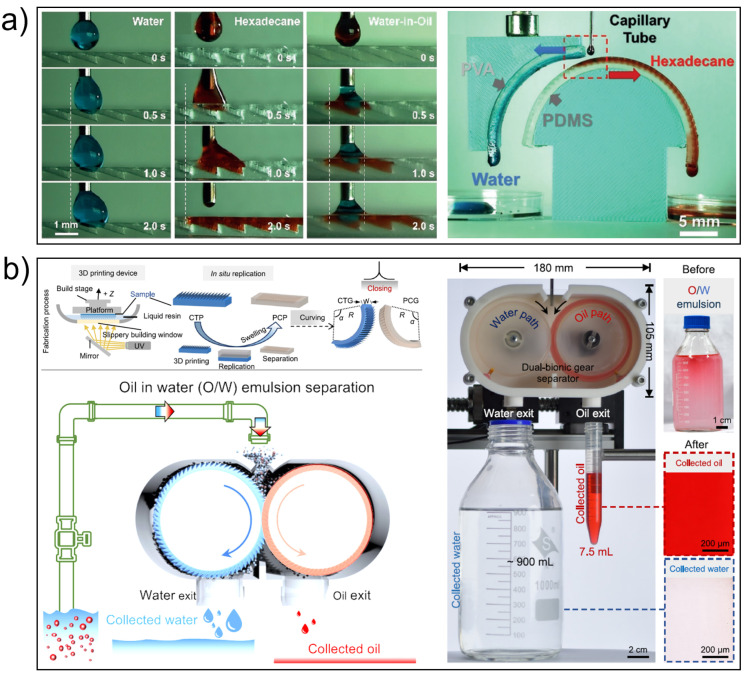
Oil/water separation on the peristome-mimetic film. (**a**) The spreading behaviors of water, hexadecane, and water-in-hexadecane drops on the peristome-mimetic PDMS surface (**left**), and the pure water and oil drops drip periodically from the opposite sides of the water–oil separation device (**right**); (**b**) Dual-bionic gears for oil–water separation. A 3D printing method and a replicating method are used to construct the bio-mimetic cat tongue surface and peristome-mimetic surface which are integrated into dual-bionic gears separator for oil-in-water (O/W) emulsion (**left**); the opaque emulsion is separated into clear water and clear oil phases after the dual-bionic gear separation (**right**). Reproduced with permission from Refs. [22,101].

***Liquid–Liquid separation*:** Bioinspired liquid–liquid separation systems have recently achieved remarkable progress through synergistic integration of natural principles and advanced manufacturing technologies. As a representative advancement, Li et al. [22] developed a peristome-mimetic surface via 3D printing technology, incorporating arch-shaped microcavities to enable spontaneous microscale water-in-oil separation through curvature-driven capillary action. This system attained nanoliter-scale separation efficiency without external energy input by leveraging hydrophobic polydimethylsiloxane (PDMS) and hydrophilic polyvinyl alcohol (PVA) dual-surface interactions, demonstrating exceptional capability in processing viscous liquids and oil-in-oil mixtures with ultralow interfacial tension differences (Δγ < 1 mN/m). Such innovations hold transformative potential for microfluidic diagnostics and environmental remediation applications (Figure 6a).

Concurrently, adaptive membrane technologies have emerged as a pivotal research direction. Zhang et al. [100] fabricated a Janus membrane through cyclic self-assembly of phytic acid-Fe^3+^ complexes combined with PDMS spray coating, achieving tunable unidirectional liquid transport (ULT) functionality. The membrane exhibited dual operational modes: ULT mode accelerated moisture evaporation rates by 160% compared to conventional cotton substrates while simultaneously demulsifying oil-in-water emulsions, while its switchable permeation mode enabled gravity-driven separation with ultrahigh throughput and >99.9% efficiency across 10 cycles. Notably, its fluorine-free fabrication protocol and mechanical resilience (withstanding 300 abrasion/ultrasonic cycles) significantly enhanced practical viability for smart textile engineering and offshore oil spill remediation.

Extending dynamic separation concepts, integrated bionic systems demonstrate unprecedented performance. Wang et al. [101] developed a dual-bionic rotary separator featuring superhydrophilic 3D-printed teeth (mimicking feline lingual papillae) and superoleophilic PDMS cavities (inspired by Nepenthes peristomes). This rotating mechanism attained >99% separation efficiency for multi-scale emulsions at 2000 L·m^−2^·h^−1^ processing flux, maintaining <3% flux decline over 600 min through autonomous mechanical self-cleaning (Figure 6b). The system demonstrated chemical stability across pH 1–13 environments and effectively processed complex kitchen wastewater (97% efficiency), addressing critical limitations of static membranes in flux sustainability and fouling resistance for industrial/marine applications.

Amphibious separation capabilities further expand operational scenarios. Inspired by Namib beetles, Li et al. fabricated nano/micro-roughened cellulose surfaces with amphiphilic wettability, achieving a 173° hexadecane contact angle and >98% separation efficiency for viscous oil–water mixtures while preserving 10 kPa air permeability [102]. Complementing these developments, Wei’s Janus membrane [103] realized bidirectional transport functionality, maintaining >98% separation efficiency under hypersaline conditions (10 wt% NaCl) through optimized pore geometry and surface charge modulation.

Collectively, these advancements underscore four evolutionary trends: biomimetic structural optimization enhancing capillary/interfacial effects; dynamic operational modalities enabling adaptive separation; scalable manufacturing protocols improving commercial feasibility; and environmental resilience expansion for extreme operational conditions. Future research directions should prioritize intelligent responsivity, energy-autonomous operation, and circular economy compatibility to address global water–energy nexus challenges.

**Figure 7:**
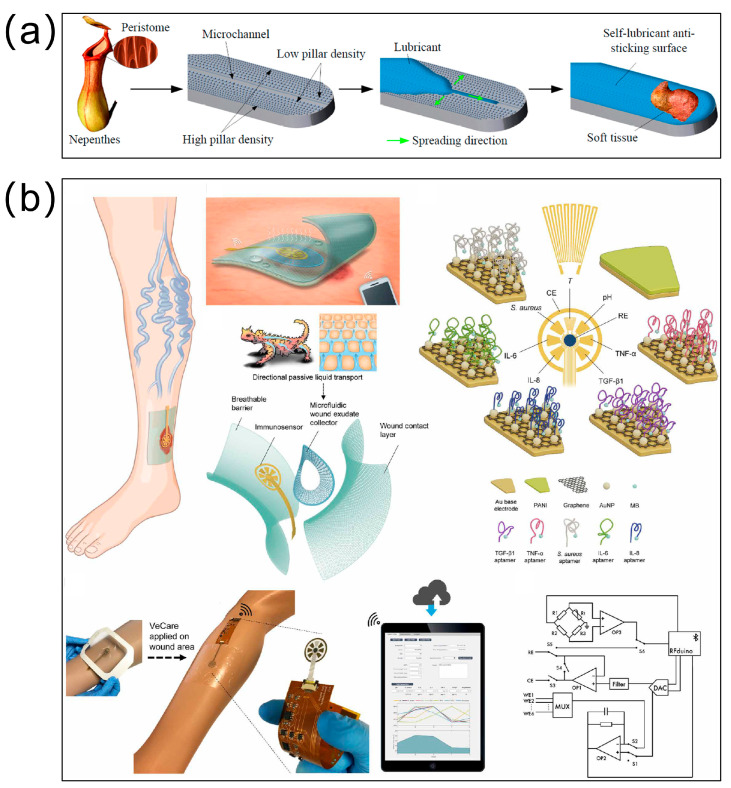
Bioinspired materials for biomedical usage. (**a**) Nepenthes−inspired self−lubricating slipery surface with engineered wettability gradients achieves enhanced anti−adhesion performance by leveraging non−uniformly arranged cylindrical micropillars on electrosurgical scalpel surfaces to drive spontaneous lubricant migration along the microstructured topography. (**b**) A multiplexed immunosensing system for chronic wound monitoring. Inspired by the skin of Texas horned lizard enabling predetermined flow direction, a microfluidic wound exudate collector is designed. The multiplexed immunosensing system, combined with a perforated wound contact layer, a microfluidic wound exudate collector, an immunosensor, and a breathable barrier can monitor a chronic wound. Reproduced with permission from Refs. [85,104].

***Biomedical***: Biomimetic engineering has catalyzed transformative innovations in biomedical fluidic systems through multiphysics-coupled architectural design. As a paradigm-shifting advancement, Deng et al. engineered a multi-gradient synergistic mechanism integrating Laplace pressure modulation, wettability gradient alignment, and wettability contrast optimization. This system enabled directional transport of 5 μL droplets over 2.6 mm on high-adhesion surfaces with inverted stability metrics, validated through predictive mechanical modeling. The breakthrough demonstrates critical potential for microreactor precision control and targeted drug delivery platforms [39]. Complementing these efforts, Liu’s team addressed soft tissue adhesion challenges in electrosurgery by fabricating photolithography-assisted wettability-gradient surfaces on 316 L stainless steel substrates. The silicophilic-modified micropillar arrays (facilitated directional silicone oil propagation at 452 μm/3.49 s, achieving 90% adhesion reduction while maintaining operational integrity through 10^3^ cyclic sterilization protocols, thereby revolutionizing minimally invasive surgical instruments [104] (Figure 7a).

Chronic wound management has been redefined through bioinspired diagnostic integration. Gao et al. [85] developed VeCare—a flexible microfluidic multiplexed immunosensing platform mimicking Phrynosoma cornutum skin microstructure. The system synergizes graphene-Au nanocomposite electrochemical sensors with an aptamer-functionalized directional exudate transport, enabling real-time monitoring of six wound microenvironment parameters (pH, TNF-α, IL-6, etc.). Wireless smartphone integration facilitates instantaneous data transmission, representing a 300% enhancement in diagnostic resolution compared to conventional single-marker assays (Figure 7b). This innovation bridges critical gaps between smart dressing technology and personalized wound therapeutics.

Clinical translation challenges are being systematically addressed through biomechanical optimization. Li’s topological liquid diode catheter employed UV-resin replicated reentrant microstructures to achieve 0.85 mm/s forward flow with complete backflow suppression, maintaining performance through 100 bending cycles, which shows promise for anti-reflux medical catheters [105]. Extending biomimetics to precision machining, Chen’s team developed femtosecond-laser-textured tools mimicking Nepenthes’ peristome. The hierarchical micro-grooves and arc pits enabled 3.7 m/s directional cutting fluid transport, reducing tool wear by 84% and achieving nanoscale surface roughness, crucial for manufacturing biomedical titanium components [106]. These breakthroughs, through innovative bioinspired architectures and multiphysics coupling mechanisms, are driving advancements from microfluidic diagnostics to smart implants, with future potential in organ-on-chip systems and precision therapeutics.

***Agriculture***: Biomimetic engineering principles have been innovatively applied to advance solar desalination technologies through synergistic architectural–thermal optimization. Contemporary bioinspired solar evaporators exhibit novel strategies to overcome hypersaline water treatment limitations by harmonizing structural design with interfacial hydrodynamic control. Wu et al. [107] engineered a biomimetic evaporator integrating avian beak capillary ratchets and Nepenthes peristome morphology. The gradient microcavity arrays generated Marangoni-effect-induced aqueous films that spatially confined salt crystallization to designated zones, sustaining >96% energy efficiency under 25 wt% NaCl concentration. This system attained an evaporation rate of 2.63 kg·m^−2^·h^−1^ under 1 sun irradiation while enabling mechanical salt removal compatibility, demonstrating exceptional resilience in hypersaline environments.

**Figure 8:**
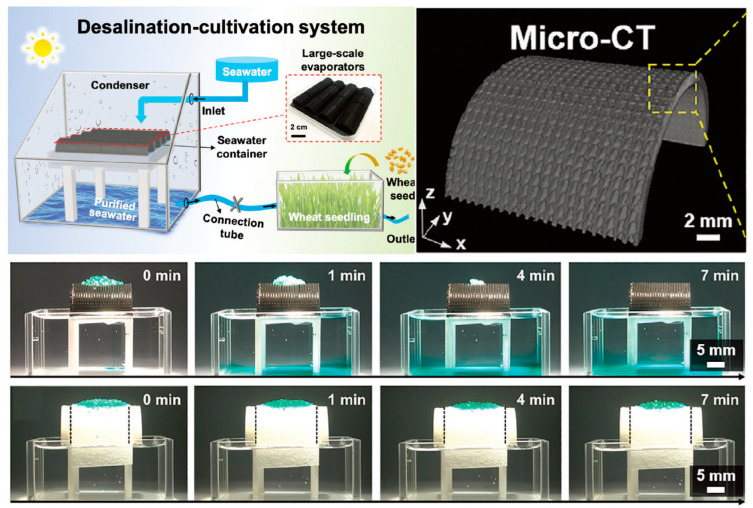
A biomimetic Bridge-Arch solar evaporator for eliminating salt accumulation with desalination. The 3D bridge-arch evaporator with peristome-mimetic microstructures is fabricated by a Digital Light Processing additive manufacturing process; and time sequences of optical images characterize the rejecting process of 0.1 g NaCl solid crystal from the bridge top of the bridge-arch evaporator to bulk water within 7 min, and the insufficient salt-rejecting process of 0.1 g NaCl solid crystal from the bridge top of filter paper to bulk water. Reproduced with permission from Ref. [49].

Complementing these structural innovations, Zou et al. [49] developed a bridge-arch configuration featuring a dual-layer liquid film mechanism. This architecture synergized restricted water supply channels with free-flowing Marangoni convection, effectively mitigating salt accumulation under 20 wt% brine conditions (Figure 8). Fabricated via UV-cured composite materials, the system maintained sustained desalination performance over 200 operational hours while producing agriculturally compliant water. These advancements collectively transcend conventional evaporation limitations through two distinct bioinspired thermal management paradigms: System 75 enables continuous darkness operation by leveraging residual thermal gradients, whereas System 84 couples structural durability with agricultural irrigation compatibility.

The integration of three-dimensional biomimetic architectures has established new benchmarks in desalination technology. These designs achieve an optimal balance between superior flux, extreme salinity tolerance, and scalable fabrication through additive manufacturing and resin-refilling techniques. Notably, the biomimetic 3D configurations facilitate multiscale water–energy–food nexus solutions by simultaneously addressing freshwater production, energy efficiency, and agricultural sustainability requirements.

## 7. Conclusions

In this review, we systematically summarize the directional liquid transport on planar surfaces in a passive and energy-free way inspired by natural systems. Firstly, we systematically introduce the directional liquid transport phenomena existing in natural prototypes from nature, such as desert beetles, Texas horned lizards, and pitcher plants, which utilize sophisticated structural and chemical mechanisms for efficient liquid transport. Then, we analyze various forces driving directional liquid transport on planar surfaces which utilize a synergistic effect of chemical gradients, geometric asymmetry, and wettability heterogeneity to achieve efficient liquid manipulation without external energy input. Subsequently, we comprehensively summarize recent research advancements in biomimetic materials and devices for water harvesting and directional liquid transport, with particular emphasis inspired by natural principles. Finally, we conclude with engineering applications in smart textiles, oil–water separation, biomedical applications, and agriculture systems, highlighting their potential implementations in industrial technologies.

Biomimetic technology opens up innovative ways to solve water scarcity and sustainable energy challenges. By deeply analyzing the delicate structures formed by the evolution of organisms over millions of years, researchers have realized high water harvesting efficiency and directional transport of liquids on a planar surface. In dealing with the water resource crisis, the ultra-high water harvesting efficiency of atmospheric water in arid areas is realized by coordinating Laplace pressures and surface energy gradients. In the field of energy, the droplet collection is combined with electrostatic power generation to create a new model of water–energy co-generation. After the porous structure integrates photothermal materials, it can realize solar seawater desalination and collect fog at night, forming an all-weather water–energy symbiosis system. These technological breakthroughs reveal the key role of multistage bionic structures and multi-physical field synergies (surface tension, capillary action, pressure gradient, etc.) and are expected to build distributed resource supply networks in arid regions, providing innovative paradigms that are both ecologically smart and feasibly engineered for sustainable development goals.

## 8. Perspectives

Despite significant advancements in bioinspired directional liquid transport systems, several challenges impede their practical deployment. A primary limitation lies in scalability and durability, as the intricate micro/nanostructures mimicking natural systems remain costly to fabricate at industrial scales using current techniques like laser patterning or 3D printing. Material degradation under harsh environmental conditions further compromises long-term reliability. Additionally, while stimuli-responsive surfaces show promise for adaptive fluid control [104,105], their slow actuation speeds hinder real-time applications. Current designs also struggle to handle multiphase or non-Newtonian fluids, limiting their utility in biomedical or industrial settings where complex fluids dominate. Moreover, passive systems face inherent trade-offs between energy efficiency and performance under low-humidity or extreme temperatures.

Looking ahead, overcoming these barriers will require interdisciplinary innovation. Advanced manufacturing methods could enable scalable, cost-effective, precise fabrication of multiscale architectures. Integrating machine learning with bioinspired design may accelerate the optimization of surface geometries for target applications, while novel stimuli-responsive materials with sub-second switching capabilities could revolutionize adaptive microfluidics and wearable technologies. Finally, synergizing these innovations with sustainable infrastructure, such as solar-powered water harvesting or self-irrigating agricultural systems, could address critical global challenges in water-energy-food security. By bridging fundamental insights with scalable engineering, next-generation bioinspired fluidic systems are poised to transition from lab-scale prototypes to real-world solutions.

## Data Availability

No new data were created or analyzed in this study.
